# Undiluted Serum Eye Drops for the Treatment of Persistent Corneal Epitheilal Defects

**DOI:** 10.1038/srep38143

**Published:** 2016-12-02

**Authors:** Kaevalin Lekhanont, Passara Jongkhajornpong, Thunyarat Anothaisintawee, Varintorn Chuckpaiwong

**Affiliations:** 1 Department of Ophthalmology, Ramathibodi Hospital, Mahidol University, Bangkok, Thailand; 2 Department of Family Medicine, Ramathibodi Hospital, Mahidol University, Bangkok, Thailand

## Abstract

Several studies found that 50–100% serum eye drops provided greater benefits without inducing detrimental effects on the corneal epithelial healing. This study assessed the efficacy of undiluted serum eye drops for the treatment of persistent corneal epithelial defects (PED). A total of 109 eyes received 100% serum eye drops for PED were recruited into this study. The data were compared with an historical control group of 79 eyes with PED who received conventional treatments from 2006–2011 at the same institution. Main outcome measures were complete healing of PED and incidence of adverse events. No significant difference in demographics between the 2 groups was noted. The success rate of the treatment and control groups were 87.16% (95% CI 0.79–0.93) and 69.62% (95% CI 0.59–0.80) (*P* = 0.001), respectively. The median time to complete epithelialization was 14 days (95% CI 12–21) in the treatment group and 28 days (95% CI 21–59) in the control group (*P* = 0.001). Serum treatment, primary diagnosis of non-limbal stem cell deficiency etiology, and prior contact lens wear significantly correlated with the corneal re-epithelialization. There were no serious side effects encountered during the study period. In conclusion, undiluted serum therapy is effective and safe for treating PED.

Persistent corneal epithelial defects (PED) have been defined as an epithelial defect that does not heal within the expected time frame despite conventional treatment, which is usually described as 2 weeks in the literature^1^,^2^,^3^,^4^. Although PED are uncommon, they may lead to corneal inflammation, infection, scarring, melting, and even perforation^5^. PED can be a consequence of numerous diseases, including severe dry eye, limbal stem cell deficiency (LSCD), neurotrophic keratitis, exposure keratopathy, and post-infectious corneal ulceration, which are intractable problems and occasionally cannot be directly managed^3^,^6^. Therefore, a variety of standard therapies consisting of aggressive lubrication with preservative-free artificial tears, bandage soft contact lenses, pressure patching, epithelial debridement, and tarsorrhaphy have been used in an attempt to heal PED^2^. Nonetheless, some PED fail to resolve with these first-line treatments in an orderly and timely manner.

Human serum eye drops not only function as a natural preservative-free lubricant, but also supply epitheliotrophic factors and essential substances for the recovery of a damaged epithelium, potentially making it an excellent tear substitute^6^,^7^,^8^,^9^. Previous studies have proved the efficacy of a 20% solution in the treatment of PED, with 60–70% of eyes healing completely within 4 weeks^3^,^10^,^11^. This 1:5 dilution is empirically used to decrease the concentration of TGF-β, which is a pro-inflammatory factor, to physiologic tear levels^12^,^13^,^14^. Nevertheless, dilution could in turn reduce the concentration of other beneficial factors, particularly epidermal growth factor (EGF) and fibronectin. With the optimized manufacturing protocol, the serum and tear concentration of TGF-β are supposed to be equivalent and dilution may not be necessitated^15^. Recently, several studies found that higher concentrations (50–100%) seemed to provide greater benefits without inducing detrimental effects on the corneal epithelial healing^5^,^16^,^17^,^18^,^19^,^20^,^21^,^22^.

The purpose of this study was to evaluate the efficacy and safety of 100% serum eye drops for the treatment of PED not related to ocular surgery.

## Methods

### Study design

This was a single-center, prospective, interventional trial conducted between December 2011 and December 2015. The data were compared with an historical control group of patients with PED who received standard conventional treatment but not serum eye drops from 2006–2011 at the same institution. The study was approved by the Ethics Committee of Mahidol University School of Medicine and conducted in accordance with the Declaration of Helsinki. All participants were informed about the aim and protocol of the study and gave written informed consent prior to undergo the procedures.

### Patients

A total of 101 participants, older than 15-years-old of age, with corneal epithelial defects of at least 14 days of duration who had not responded to conventional therapy, were enrolled into the study. Conventional treatment options which were employed in both treatment and control groups included hourly preservative-free artificial tears including sodium hyaluronate eye drops, lubricating gel/ointment, anti-inflammatory agents, punctal plugs, bandage contact lenses, and discontinuation of the offending topical medications. Exclusion criteria are as follows:

- previous ocular surgery;

- having any active ocular infection;

- having progressive ulcerative keratitis caused by autoimmune diseases;

- pregnant or lactating women;

- not able to comply with the study regimen

The same eligibility criteria and evaluation procedure were applied for 76 historical controls.

### Interventions

Serum eye drops were prepared according to the standardized protocol of Geerling G *et al*. and Liu *et al*^15^,^18^. Briefly, venipuncture was performed at the antecubital fossa and 50–100 ml of whole blood was collected into sterile tubes without clot activator or anticoagulant. The tubes were left standing for 2 hours at room temperature (18–25 °C) in an upright position for complete clotting, followed by centrifugation at 3000 *g* for 15 minutes. The supernatant serum was transferred into sterile plastic tubes using single-use sterile pipettes under a laminar air flow hood. The volume retrieved was determined and filter-sterilized (0.2 μm) with no dilution. All preparations were performed by only one well-trained medical staff, using proper aseptic technique. No more than one blood sample from one person was manipulated at the same time. Allogeneic serum donated from a family member was used when a patient’s own serum was unsuitable or unavailable for processing into autologous serum eye drops^20^,^23^.

Patients were instructed to store all unopened bottles of serum in their freezer ideally at −20 °C and thaw one bottle for use at a time in the refrigerator at 4 °C. A shelf life for the thawed bottle was set at 24 hours; it was kept in the refrigerator (4 °C) after each use and discarded at the end of the day. If the domestic freezer and refrigerator had no thermometer, patients were asked to place the bottles inside and finish the eye drops within a shorter storage time^20^. The frequency of instillation was one drop every hour while awake until the defect was healed. Once complete epithelial healing occurred, the serum eye drops were gradually tapered to 4 times daily for an additional 2 weeks and then discontinued.

During administration of serum eye drops, the preservative-free artificial tears including sodium hyaluronate drops and contact lenses were not used. The patients were evaluated every day for admitted patients and every 1 to 2 days for outpatients until there was a total corneal epithelialization. On days 7, 14, 21, 28, and then every 1 month after weaning of serum therapy, all patients were reexamined for any recurrences or new epithelial defects, and complications. They were followed up for a minimum of 6 months. Slit-lamp biomicroscopic examination with fluorescein staining was performed at baseline and all follow-up visits. The size of the epithelial defect was measured in two linear dimensions, the longest linear diameter and the largest one perpendicular to it, within the confines of the PED and calculated by multiplying the two linear dimensions^24^.

### Main outcome measures

Primary outcome measures were complete healing of the PED and incidence of adverse events. Secondary outcomes were time to complete corneal epithelialization, the relationship between subject characteristics and outcomes, and recurrence of the PED after closure. Success was defined as the complete closure of PED. Failure was defined if (a) there was no objective improvement in corneal epithelial healing within 1 month of serum therapy, (b) the lesion was enlarging or worsening, or (c) surgical intervention was necessary. Recurrence or new epithelial defect was defined as a corneal epithelial defect that occurred at the same site or different area within 1 month after initially complete epithelialization respectively.

### Statistical analyses

Statistical analyses were performed with the statistical software package STATA version 14 (Stata Corp, College Station, Texas, USA). Baseline characteristics of the 2 groups (treatment and historical control) were compared using independent t-test, chi-square and Fisher’s exact tests. Mean and standard deviation (SD) or median and range were used to describe continuous data. Frequency and percentage were used for categorical data. Kaplan–Meier survival analysis was used to estimate the success rate and overall probability of complete corneal epithelialization occurring at different time points. Log-rank test and Cox proportional hazards regression analysis were applied to assess the independent association between predictor variables and complete corneal epithelialization. Variables with *P* < 0.10 in univariate analysis were selected for Cox regression multivariate analysis. A *P* < 0.05 was considered to be statistically significant.

## Results

The mean age of the treatment group was 45.4 ± 19.3 years (range, 16–82 years) and 57.8% were male. In the control group, the mean age was 53.7 ± 14.1 years (range, 26–81 years) and 45.6% were male. Most patients in both groups (53.2% of the treatment group and 65.8% of the control group; P = 0.16) lived in the urban areas and suburbs of Bangkok. There were no significant differences in baseline characteristics between the 2 groups (Table 1). Of the 109 eyes in the treatment group, 104 eyes (95.41%) received autologous serum eye drops and 5 eyes (4.59%) received allogeneic serum eye drops. The reasons for using allogeneic serum eye drops in some cases included hepatitis B virus (HBV) carrier (4) and human immunodeficiency virus (HIV) infection (1). Allogeneic serum was taken from the patient’s spouse (3) and offspring (2). The donors were tested for blood-borne diseases such as HIV, hepatitis B and C virus and syphilis using standard blood bank screening tests to ensure that their serum was suitable for processing into serum eye drops. The duration of the PED prior to the commencement of serum eye drops was 14 days in all cases. The mean area of PED was 35.65 ± 19.74 mm^2^ (range, 2–110.25 mm^2^). Approximately 30% of treatment patients did not wear bandage soft contact lenses prior to serum treatment mainly because of a high risk of superinfection (17 eyes, 47.22%), an inability to have a comfortably well-fitting contact lens (12 eyes, 33.33%), and continuous falling out of contact lens (7 eyes, 19.44%). The mean follow-up was 64.88 ± 18.16 months (range, 45–80 months).

**Figure 1:**
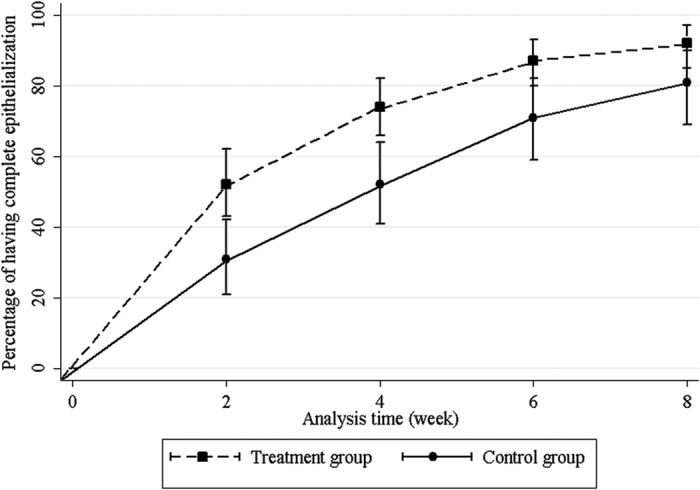
Probability of having complete corneal epithelialization at each time point in both treatment and control groups.

The overall success rate of the treatment and control groups were 87.16% (95/109 eyes; 95% CI 0.79–0.93) and 69.62% (55/79 eyes; 95% CI 0.59–0.80) (*P* = 0.001), respectively. The median time to complete corneal epithelialization was 14 days (95% CI 12–21) in the treatment group and 28 days (95% CI 21–59) in the control group (*P* = 0.001) (Fig. 1). Univariate analyses demonstrated that serum treatment and primary diagnosis of non-LSCD etiology had significant positive correlations with corneal re-epithelialization. The prior bandage contact lens wear was the only variable negatively correlated with corneal re-epithelialization (Table 2). From the multivariate model, these 3 factors remained significantly correlated with the corneal re-epithelialization (Table 3). Fourteen eyes in treatment group (12.84%) and 24 eyes (30.38%) in control group required surgical interventions (Table 4). In treatment group, recurrence of the corneal epithelial defect after initially complete closure and stopping serum eye drops occurred in 2 eyes with chemical injury, 2 eyes with contact lens-induced LSCD, 1 eye with postinfectious persistent epithelial defect, 2 eyes with Stevens-Johnson syndrome (SJS), and 1 eye with exposure keratopathy. New corneal epithelial defects were developed in 1 eye with chemical injury and 1 eye with mucous membrane pemphigoid (MMP) while weaning of serum therapy. All recurrent and new lesions were smaller than the original ones. The patients were successfully retreated with 100% serum eye drops.

Adverse reactions were observed in 2 patients (1.83%) receiving autologous serum eye drops. One patient had non-painful red swollen eyelids every time he had been given the 100% serum eye drops (1 drop hourly). Eyelid swelling was not accompanied by itching or burning. He was sent for a consultation with an immunologist and suspected to have an allergic reaction to his own serum eye drops. Hence, the serum treatment was discontinued. The redness and swelling were gone within 72 hours of cessation of the medication. He was one of the three patients with postinfectious persistent epithelial defects who had treatment failure in Table 4. The patient had an underlying Bell’s palsy with incomplete recovery and finally needed amniotic transplantation and tarsorrhaphy to heal the PED. Due to the patient’s financial hardship, the true cause of the allergic reaction was not investigated. Another patient had sticky sensation with mild ocular discomfort but willingly continued treatment. No any side effects were seen in patients receiving allogeneic serum eye drops. Also, there were no serious complications such as infectious keratitis encountered during the entire study period.

## Discussion

The application of serum eye drops has gained popularity as a second-line therapy in the treatment of ocular surface diseases. Nonetheless, the optimal treatment concentration of serum eye drops has not been established. Because of the limited efficacy noted with 20% and 50% serum eye drops at our institution plus inconsistent effectiveness of diluted serum in improving dry eye symptoms and signs reported in previous studies, we chose to use 100% serum^23^. Undiluted serum eye drops are also asserted to provide higher concentration of growth factors as well as avoid possible toxicity of diluents and any contamination during the dilution process^20^. Additionally, as the defect duration is the most useful predictor of healing time^5^, we started serum treatment 2 weeks after patients had unresponsive PED, which was earlier than previous studies^3^,^5^,^10^,^11^,^16^, in order to minimize the healing time and chances of chronic epithelial defect-related complications.

Our study demonstrated higher success rates and shorter re-epithelialization time of 100% serum eye drops in patients with PED compared to conventional treatments. Serum treatment, primary diagnosis of non-LSCD etiology and prior bandage contact lens wear were the major variables correlated with the corneal re-epithelialization. Despite early treatment with 100% serum, our favorable results are not remarkably superior to those of all previous studies using lower concentration serum eye drops (Table 5). Considering that most of these studies are non-comparative case series and run different protocols for the production of the serum, therefore besides applying different serum concentration, a variety of steps of serum production, including clotting time, centrifugation time and force, dilution, and diluents might yield various concentrations of the epitheliotrophic factors in the resulting serum even in the same serum concentration. In addition, there are many other different factors among these studies such as indications, size and duration of the lesion, and serum dosage regimen which might variably impact the outcomes as well, making it more difficult to directly quantitatively compare the outcomes between these studies. The reasons for this observation may be the original diagnosis which excluded corneal epithelial defects after ocular surgery and the relatively larger size of the PED in our patients. Generally, non-surgical PED tend to have predisposing ocular surface problems with poorer wound healing but most postoperative PED could finally heal in an acceptable time frame because of their less complicated ocular conditions^20^. Accordingly, the high success rate in our study is likely due to the use of undiluted serum eye drops. Another interesting point is that the study from Poon *et al*. is the most similar to this study in relation to the serum concentration with small number of post-operative indications, however, it had the smallest percentage of success rate compared to the others that used 20% of serum concentration. This might be explained by the longer duration of PED plus a less frequent dosing regimen in the series of Poon *et al*. Furthermore, there have been 2 previous studies showing that 100% serum eye drops clinically produced significantly faster epithelial would closure than 50% serum eye drops^16^,^19^. The undiluted autologous serum has also been demonstrated to be more effective than 20% serum in the epithelial healing process of mechanical corneal ulcers in animal study^17^. Additionally, 100% serum resulted in better human epithelial cell migration than diluted one, probably because of the higher concentration of fibronectin^18^.

Although the off-label use of topical 100% serum eye drop appeared to be successful, this approach could not overcome the basic underlying pathology of various diseases as observed in some of our patients with preexisting definite ocular surface disorders such as severe neurotrophic keratopathy, exposure keratopathy, and particularly total LSCD due to alkali injuries, MMP, SJS, or toxic epidermal necrolysis (TEN) who either failed to serum treatment or developed recurrent or new lesions after initially complete reepithelialization. Therefore, primary correctable pathologies should be addressed along with the serum therapy if possible to permanently heal PED. Also, as limbal epithelial stem cells have a relatively long cell cycle time, longer serum therapy may be needed to allow more time for an improvement in the ocular surface microenvironment and healing of PED to occur.

It is interesting that, in our study, prior contact lens wear had a negative influence on corneal re-epithelialization. One possible explanation for this finding is that bandage contact lens had been used in only patients with a high degree of disease severity because our patients were prone to get keratitis from contact lenses owing to a warm, humid climate and under-education about infections secondary to contact lens use. Thus, prior contact lens use might represent a more severe disease, indirectly increasing the vulnerability to poor outcomes.

Regarding safety issues, one patient in our series had a presumptive ocular allergy to his own serum. To the best of our knowledge, this has never been reported in the literature. Unfortunately, additional work-up to find the exact etiology were not undertaken because of the patient’s financial constraints. Moreover, the downside of using 100% serum eye drops includes patient inconvenience of large volume of blood collection, the likelihood of repeated blood draws, and an economic burden on patients.

The limitations of this study included inherent shortcomings in any historical control study, heterogeneous patient populations, and short follow-up time. Since serum eye drops which has been considered a potential effective treatment for PED is available although off-label use, the use of a placebo control group in this trial would cause an ethical problem, as it requires some patients to go without useful treatment. However, the absence of a concurrent placebo group limits the interpretation of the therapeutic effect of 100% serum eye drops. Using the control data from other previous studies might not match for characteristics important to corneal epithelial healing as well. Hence, use of a historical control appears to allow the further assessment of the effect of 100% serum eye drops on PED in the active-control study without placebo group. The major disadvantage of the historical control approach is the lack of randomization. Additionally, although the control group received the same conventional treatment options as the current ones in the treatment group, the dosage regimen and the brand of medications were not identical. Because of retrospective data collection, there was also no sufficient quantitative information about the severity of the control group, for example, the duration or the mean area of the PED. Nevertheless, the similarity in overall baseline characteristics of the treatment and control groups ensures that the 2 groups may be acceptably balanced with regard to risk factors and partially protected against selection bias. Despite our study being conducted at the large ophthalmology referral center, we faced challenges in recruiting homogeneous patient groups since PED was uncommon and could arise from a number of causes. Subsequently, comparison of different PED’s sizes and etiologies between treatment and control groups can still result in selection bias. Future long-term studies comparing not only the 100% concentration with placebo, but also with the other more commonly used concentrations (20 and 50%) are necessitated to fully understand the effects of human serum on each stage of corneal wound healing, to determine the optimum concentration of serum for specific diseases as well as adverse reactions and recurrences of PED for some certain condition, and to properly implement serum eye drops as a standard treatment modality.

In conclusion, on the basis of the analysis using matched historical controls, 100% serum eye drops are very effective, safe, and tolerable for treating PED. It could be a therapeutic option with the potential benefits of quickly healing defect, preventing complications from unhealed ulcers, and reducing the need for invasive ocular surgical interventions, frequent patient follow-up, and hospitalization.

## Additional Information

**How to cite this article**: Lekhanont, K. *et al*. Undiluted Serum Eye Drops for the Treatment of Persistent Corneal Epitheilal Defects. *Sci. Rep.* **6**, 38143; doi: 10.1038/srep38143 (2016).

**Publisher's note:** Springer Nature remains neutral with regard to jurisdictional claims in published maps and institutional affiliations.

## Figures and Tables

**Table 1:** Participant baseline characteristics demonstrating that there were no significant differences in patient demographics between the treatment and historical control groups.

Characteristics	Treatment (n = 109 eyes) (%)	Control (n = 79 eyes) (%)	*P* value
Age (years): Mean ± SD (Range)	45.4 ± 19.3(16–82)	53.7 ± 14.1(26–81)	0.13
≤40 years	18 (16.5)	7 (8.9)	
>40 years	91 (83.5)	72 (91.1)	
Sex:			0.26
Male	63 (57.8)	36 (45.6)	0.47
Female	46 (42.2)	43 (54.4)	0.2
Diagnosis			0.39
-Chemical/thermal injuries	29 (26.6)	18 (22.8)	
-Neurotrophic keratopathy	25 (22.9)	30 (38.0)	
-Postinfectious persistent epithelial defects	15 (13.8)	9 (11.4)	
-Stevens-Johnson syndrome/toxic epidermal necrolysis	11 (10.1)	4 (5.1)	
-Bullous keratopathy	6 (5.5)	3 (3.8)	
-Contact lens-related limbal stem cell deficiency	6 (5.5)	2 (2.5)	
-Recurrent corneal erosion	5 (4.6)	3 (3.8)	
-Vernal keratoconjunctivitis with shield ulcer	4 (3.7)	2 (2.5)	
-Mucous membrane pemphigoid	3 (2.6)	1 (1.3)	
-Exposure keratopathy	3 (2.6)	1 (1.3)	
-Graft-versus-host disease	1 (0.9)	1 (1.3)	
-Unknown cause limbal stem cell deficiency	1 (0.9)	—	
-Severe dry eye	—	4 (5.1)	
-Aniridia keratopathy	—	1 (1.3)	
Diabetes mellitus	34 (31.2)	21 (26.6)	0.47
Prior contact lens use	73 (67.0)	45 (57.0)	0.020

n = number, SD = standard deviation.

**Table 2:** Univariate analysis of the association between each patient variable and outcomes showing that primary diagnosis of non-limbal stem cell deficiency, prior bandage contact lens wear, and serum treatment had significant correlations with corneal re-epithelialization.

Patient variables	Success (%)	Failure (%)	Hazard ratio	95% CI	*P* value
Age					
≤40 years	11.9	18.9			
>40 years	88.1	81.1	1.18	0.72–1.94	0.51
Sex					
Male	48.3	56.8			
Female	51.7	43.2	1.31	0.95–1.82	0.10
Diagnosis					
Limbal stem cell deficiency	37.1	73			
Non- limbal stem cell deficiency	62.9	27	1.71	1.22–2.39	0.002^†^
Diabetes mellitus					
Absent	86.7	91.9			
Present	13.3	8.1	0.300.77	0.48–1.26	0.30
Prior contact lens use					
No	59.6	40.5			
Yes	40.4	59.5	0.64	0.46–0.90	0.01^†^
Serum treatment					
No	37.1	62.2			
Yes	62.9	37.8	1.72	1.22–2.42	0.002^†^

^†^*P* < 0.10, CI = confidence interval.

**Table 3:** Multivariate analysis of the association between patient variables and outcomes showing that primary diagnosis of non-limbal stem cell deficiency, prior bandage contact lens wear, and serum treatment remained significantly correlated with the corneal re-epithelialization.

Patient variables	Hazard ratio	95% CI	*P* value
Serum treatment	1.80	1.7–2.54	0.001**
Diagnosis of non-limbal stem cell deficiency	1.64	1.16–2.30	0.005**
Prior contact lens use	0.65	0.46–0.91	0.012*

The prior bandage contact lens wear was the only variable negatively correlated with corneal re-epithelialization. *P < 0.05, ** *P* < 0.01, CI = confidence interval.

**Table 4:** Summary of 38 eyes required surgical interventions.

Diagnosis	Treatment (n = 14) (%)			Control (n = 24) (%)	
	Number (%)	Serum type	Further treatment	Number (%)	Further treatment
Chemical/thermal injuries	7 (50.0)	ASE	AMT (7), tarsorrhaphy (3), LSCT (1)	10 (43.5)	AMT (10), cyanoacrylate tissue adhesive (3)
Postinfectious persistent epithelial defects	3 (21.4)	ASE	AMT (3), tarsorrhaphy (1), cyanoacrylate tissue adhesive (1), lamellar keratoplasty (1)	8 (34.8)	AMT (6), cyanoacrylate tissue adhesive (2), penetrating keratoplasty (1)
Stevens-Johnson syndrome	2 (14.3)	ASE	AMT (2)	2 (8.3)	AMT (2), tarsorrhaphy (1), lamellar keratoplasty (1)
Toxic epidermal necrolysis	1 (7.1)	ASE	AMT	1 (4.2)	AMT (1), tarsorrhaphy (1)
Postherpetic neurotrophic keratopathy	1 (7.1)	AlloSE	AMT, keratoplasty	—	—
Exposure keratopathy	—	—	—	2 (8.7)	Tarsorrhaphy (2)
Mucous membrane pemphigoid	—	—	—	1 (4.3)	AMT

All of these patients with refractory PED underwent amniotic membrane transplantation. Tarsorrhaphy was also performed in some cases. Cyanoacrylate tissue adhesive and/or lamellar or penetrating keratoplasty were done in patients with impending or frank corneal perforation.

PED = persistent corneal epithelial defects, n = number, ASE = Autologous serum eye drops, AlloSE = Allogeneic serum eye drops, AMT = Amniotic membrane transplantation, LSCT = Limbal stem cell transplantation.

**Table 5:** Comparison of the success rates of healing of PED using serum eye drops with various concentrations.

Study, year	Study design	No. of eyes	Indications (% of post ocular surgery)	Size of PED (mean ± SD, mm^2^)	Duration of PED (weeks)	Type of serum used	Concentration of serum	Frequency of serum	Success rate of healing within 4 weeks (%)
Tsubota *et al*.^7^	Prospective, non-comparative case series	16	25 (post PK)	Not reported	28.8	Autologous	20%	6–10x a day	62.5
Poon *et al*.^16^	Prospective cohort	15	6.7 (post PK)	17.7 ± 23.8	6.9	Autologous	50–100%	8x a day	46.7
Young *et al*.^10^	Retrospective, non-comparative case series	10	10 (post PK)	Not reported	3.2	Autologous	20%	6–14x a day	60
Movahedan *et al*.^11^	Prospective, non-comparative case series	20	35 (post PK, post PPV, post LASEK)	10.3 ± 11.2	7.5	Autologous	20%	6x a day	70
Jeng *et al*.^5^	Retrospective, non-comparative case series	25	36 (post PK)	Not reported	13.9	Autologous	50%	Every 2 hrs while awake	68
Current study	Prospective, historical controlled	109	none	35.7 ± 19.7	2	Autologous, allogeneic	100%	Every 1 hr while awake	73.4

Besides applying different serum concentration, there was a variety of indications, size and duration of the lesion, and serum dosage regimen among these studies. These several different factors might variably impact the outcomes as well.

SD = standard deviation, PED = persistent corneal epithelial defects, PK = penetration keratoplasty, PPV = pars plana vitrectomy, LASEK = laser-assisted subepithelial keratectomy.
